# Comparing the Effectiveness and Adherence between Fixed and Non-Fixed Dorzolamide/Timolol Maleate in Open-Angle Glaucoma Patients in Hospital Universiti Sains Malaysia

**DOI:** 10.21315/mjms2023.30.3.9

**Published:** 2023-06-27

**Authors:** Noor-Khairul Rasid, Shelva Meena Gurusamy, Liza-Sharmini Ahmad Tajuddin, Azhany Yaakub

**Affiliations:** 1 Department of Ophthalmology and Visual Science, School of Medical Sciences, Universiti Sains Malaysia, Kelantan, Malaysia; 2 Department of Ophthalmology, Hospital Universiti Sains Malaysia, Kelantan, Malaysia; 3 Department of Ophthalmology, Hospital Raja Perempuan Zainab II, Kelantan, Malaysia; 4 Department of Ophthalmology, Hospital Sultanah Bahiyah, Kedah, Malaysia

**Keywords:** dorzolamide, timolol, fixed and non-fixed, intraocular pressure, open-angle glaucoma, adherence

## Abstract

**Introduction:**

Glaucoma is an irreversible chronic eye disease in which intraocular pressure (IOP) control is important. This study aimed to assess the IOP-lowering effects and adherence scores between fixed combination dorzolamide/timolol maleate (FCDT) and non-fixed combination dorzolamide and timolol XE (NFDT) in open-angle glaucoma (OAG) patients.

**Methods:**

A randomised controlled trial in a parallel, single-blinded study involving 60 OAG patients was conducted. The patients were randomised into FCDT or NFDT based on a block randomisation technique. A pre-study run-in with Gutt timolol was administered for two weeks. IOP was assessed at baseline, month 1 and month 3, with a bottle weight measurement at month 3.

**Results:**

Only 55 OAG patients were analysed, with 8.4% dropping out. A statistically significant mean IOP reduction was observed in each group from baseline to month 1 (FCDT: mean difference [MD] = 4.93, 95% confidence interval [CI] = 4.00, 5.86); NFDT: MD = 4.92, 95% CI = 4.024, 5.82) and from baseline to month 3 (FCDT: MD 5.17, 95% CI = 4.19, 6.15; NFDT: MD = 4.85, 95% CI = 3.874, 5.82). The overall FCDT mean IOP was significantly lower by 1.02 mmHg (95% CI = −2.01, −0.02) than NFDT (*F*(1, 53) = 4.19; *P* = 0.046). A significant interaction was observed between time and treatment at month 3, with the mean IOP for FCDT being lower by 1.22 mg than for NFDT (*P* = 0.037). The mean adherence score was significantly higher in the FCDT group than in the NFDT group (*t* stat (df) = 3.88 (53); *P* < 0.001). The reduction in IOP between the groups became non-significant after adherence was adjusted (*F*(1, 52) = 2.45; *P* = 0.124).

**Conclusion:**

Both drugs showed a decrease in IOP but more so in FCDT. However, no difference was found in terms of medication adherence. An emphasis on treatment compliance is needed.

## Introduction

Glaucoma is a chronic eye disease presenting with progressive optic neuropathy accompanied by changes in the optic nerve head and retinal nerve fibre layer corresponding to visual field defect (1). According to the National Eye Survey II conducted in Malaysia, glaucoma is the third leading cause of blindness, causing 6.6% of total blindness in Malaysia (2). Glaucoma was projected to affect 64.3 million people in 2013 and 112 million by 2040 (3, 4). Malaysia is expected to be an ageing nation by year 2040, with the elderly comprising 14.5% of the estimated population of 41.5 million (5). The glaucoma burden on this population will be of significance.

The goal of glaucoma treatment is to preserve the quality of life of patients at a sustainable cost. The estimated mean cost for treatment over a lifetime is £3,001 with an annual mean cost of £475, which is burdensome on the healthcare system (6). The management of glaucoma mainly involves the instillation of topical anti-glaucoma medication, which helps in the reduction of aqueous formation, increasing outflow and neuroprotection. Managing intraocular pressure (IOP) optimally is an independent factor in disease progression. There are multiple anti-glaucoma medications available, namely prostaglandin analogues, alpha-adrenergic group, beta-adrenergic group, parasympathomimetic agents and carbonic anhydrase inhibitors (7). Studies have shown that using more than two anti-glaucoma drugs is required by many patients for IOP reduction and halting the progression of the disease (7). In comparing the concomitant usage of multiple anti-glaucoma drugs to a fixed-combination drug, the latter has shown simplicity in drug administration, reduction of drug washout and side effects from the preservatives and a better IOP reducing profile (8).

This study aimed to examine the efficacy of IOP-lowering ability and adherence scores between fixed combination dorzolamide/timolol maleate (FCDT) and non-fixed combination dorzolamide and timolol XE (NFDT) in open-angle glaucoma (OAG) patients. The fixed combination drug was dorzolamide/timolol maleate (Cosopt), while the non-fixed combination drugs were dorzolamide (Trusopt) and timolol maleate (Timoptol XE). The adherence score comparing the FCDT and NFDT groups was also assessed.

## Methods

### Study Design

This work was a single-centre, single-blinded study involving two parallel groups conducted over a period of 2 years. The study design was reviewed and approved by the Research and Ethics Committee (Human) of Universiti Sains Malaysia (USM). The study was conducted in accordance with the Declaration of Helsinki for Human Research. All patients provided their written informed consent before enrolment.

### Participants

The study participants were patients who came to the ophthalmology clinic of USM Hospital, aged more than 40 years old, with pre-existing primary OAG, either unilateral or bilateral. For bilateral involvement, the default right eye was used for the study. Patients with primary OAG, using either mono or dual anti-glaucoma therapy and with an IOP less than 35 mmHg were included. Those with secondary OAG, IOP more than 35 mmHg on dual therapy, known hypersensitivity to benzalkonium chloride or sulphonamide and known contraindication to beta blockers such as chronic obstructive pulmonary disease, asthma, bradycardia and second- or third-degree heart block were excluded.

### Procedure

**Figure 1:**
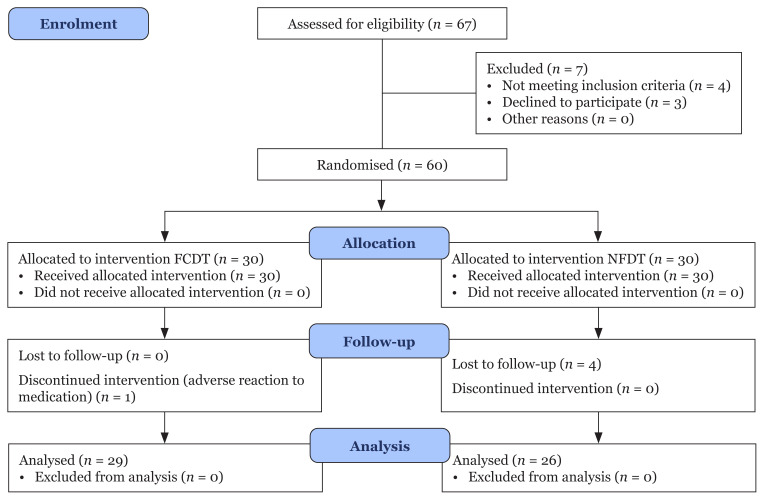
Consort flow diagram

During the first screening, a complete ocular examination was performed and the IOP was measured. The patients were asked to stop all anti-glaucoma medication and given 2 weeks of a pre-study run-in with Gutt timolol maleate 0.5% instilled at 8 a.m. and 8 p.m. daily. After 2 weeks, the baseline IOP was measured. Only those with IOP less than 35 mmHg were recruited. A block randomisation method was used to create two groups: the FCDT (group A) and the NFDT (group B). A randomised block of four was chosen, with a balanced combination of six (AABB, ABAB, ABBA, BAAB, BABA and BBAA). A block was randomly chosen using random numbers 1–6 to determine the assignment of all 60 participants. This procedure resulted in 30 participants in each group (Figure 1).

The patients in group A (FCDT) were given Gutt Cosopt (Merck & Co., Inc.) to be used twice daily at 8 a.m. and 8 p.m. The patients in group B (NFDT) were given Gutt Timoptol XE (Merck & Co., Inc.) to be instilled once daily at 8 a.m. and Gutt Trusopt (Merck & Co., Inc.) three times daily at 7 a.m., 3.30 p.m. and 11.00 p.m. Discrepancy of timing from the schedule was allowed for up to 1 h. A staff nurse gave the medication to the subjects assigned to group A and group B, and the investigators were masked. The patients were given a scheduled appointment at months 1 and 3. During the follow-up visit, visual acuity and slit-lamp examinations were conducted. IOP was measured using Goldmann applanation tonometry.

### Efficacy Assessment

The primary end point was the mean IOP change from the baseline. IOP was measured in the clinic at the baseline measurement and at the end of months 1 and 3. The readings were taken between 8 a.m. and 11 a.m.

### Adherence Assessment

At the end of month 3, the weight of the medication bottles was documented. The adherence score based on the bottle weight was obtained depending on the percentage of the medication used during the follow-up review and scoring done, as shown in Table 1.

### Statistical Analysis

Statistical analysis was performed using the IBM Statistical Package for the Social Sciences (SPSS) version 27.0. Independent *t*-test, Pearson’s chi-squared test, Fisher’s exact test, repeated measures analysis of variance (RM ANOVA) and repeated measure analysis of covariance (RM ANCOVA) were used for analysis.

## Results

A total of 60 patients were recruited, with an 8.3% dropout rate. Thus, the study was continued with 55 patients divided into the FCDT group (*n* = 29) and the NFDT group (*n* = 26). The dropout was due to a loss to follow-up of four patients and one patient experiencing a burning and stinging sensation. The mean age was 65 years old for the FCDT group and 68.5 years old for the NFDT group. Most of the patients were of Malay ethnicity. The baseline comparison between the groups was not statistically significant (Table 2).

The percentage of patients with primary OAP was 87.1% (*n* = 27) in the FCDT group and 82.8% (*n* = 24) in the NFDT group. The percentage of those with normotensive glaucoma was 12.9% (*n* = 4) in the FCDT group and 17.2% (*n* = 5) in the NFDT group. More than half of the patients had involvement of the right eye. Pre-existing anti-glaucoma medications were mainly timolol in the FCDT group (38.7%) and latanoprost in the NFDT group (58.6%).

### Efficacy

The mean IOP at baseline was 19.4 mmHg in the FCDT group and 20.7 mmHg in the NFDT group. A significant difference was found in the mean IOP based on time using RM ANOVA (*F*(2, 52) = 203.03; *P* < 0.001). Conversely, no significant difference was found in the mean IOP in each treatment group based on time after controlling for adherence using RM ANCOVA (*F*(2, 52) = 1.01; *P* = 0.341). A pairwise comparison with a confidence interval (CI) adjustment was performed. The results showed significant differences in all comparisons except between months 1 and 3 in both treatment groups in both analyses (Table 3).

A significant difference was found in the mean IOP of the two treatment groups regardless of time using RM ANOVA (*F* stat (df) = 4.19 (1, 53); *P* = 0.046) (Table 4). No significant difference was found in the mean IOP of the two treatment groups regardless of time after controlling for adherence using RM ANCOVA (*F* stat (df) = 2.45 (1, 52); *P* = 0.124).

No significant difference was observed in the mean IOP of the two treatment groups based on time using RM ANOVA (*F* (2, 52) = 0.44; *P* = 0.650) and RM ANCOVA (*F* (2, 51) = 0.90; *P* = 0.412). A pairwise comparison with a CI adjustment was performed. The results showed no significant differences in all comparisons except at month 3 (*P* = 0.037) (Table 5).

### Adherence

The bottle’s weight showed a significant weight difference between the groups (*P* = 0.044). A significant difference was found in the mean adherence score between the groups (*t* stat (df) = 3.88 (53); *P* < 0.001). The mean adherence score was higher in the FCDT group than in the NFDT group (Table 6).

## Discussion

The patients in the FCDT group had a better IOP-lowering profile with a significant and higher mean IOP reduction than the NFDT group, consistent with the findings of Shedden et al. (9) The adherence scoring using the bottle weight measurement at the end of month 3 showed FCDT having a better score.

Before the study began, all patients were given Gutt timolol 0.5% twice daily for two weeks as the run-in period to ensure a standardised baseline and a correct assessment of the efficacy of the new medication, as the insufficient washout of previous medications could lead to inaccurate results (10). After the two-week run-in, the baseline IOP was 19.4 mmHg in the FCDT group and 20.7 mmHg in the NFDT group, consistent with Francis et al. (11).

FCDT is the first combination of IOP-lowering agents recognised by the US Food and Drug Administration. As a combination of a carbonic anhydrase inhibitor and a selective b-blocker, it has a synergistic effect, working along a similar mechanism but through different pathways (12, 13). Studies have shown a reduction of about 32.7% (9 mmHg) at its peak and 27% (7.7 mmHg) throughout (14).

Dorzolamide (carbonic anhydrase inhibitor) inhibits the enzyme catalysing the hydration of CO_2_ to bicarbonate and protons in ciliary processes, thus reducing the amount of available bicarbonate and sodium ions and the production of aqueous humour (14). Dorzolamide alone gives about 17%–32% of IOP reduction. Timolol maleate is a propanolamine derivative and a non-selective beta receptor blocker. It reduces the production of the cyclic adenosine monophosphate, thus reducing the active ion transport and the production of the aqueous humour by 20%–28% (12, 14). Additive diurnal IOP reduction with dorzolamide has better IOP reduction ability at night, corresponding to 20%–23% of reduction, and Timolol has greater IOP reduction ability during the day (15, 16).

Therefore, the overall profile of the mean IOP reduction with FDCT showed a reduction of 25%–32%, with a consistent 24-h reduction and a narrow fluctuation of the IOP. The 24-h peak IOP was an independent factor for the progression of glaucoma, and those with glaucoma progression over 5 years had a significantly higher 24-h mean IOP (13).

Fixed combination therapy has been shown to be superior to the instillation of its concomitant agents. It enhances the convenience of single-bottle usage in multiple bottles and improves tolerability, as most topical anti-glaucoma medications have preservatives such as benzalkonium, which can affect ocular surface health in the long term. Therefore, this convenience also increases adherence, and the additive effect of the two medications allows the patient to attain greater IOP reduction (13). There is also a significant effect from the elimination of the washout effect of the second medication, thus allowing better absorption and more optimal drug efficacy. Holló et al. (17) found that the anatomical ability of the inferior conjunctival sac to retain medication was about 7 μL. With more medications prescribed in separate bottles, there would be wastage of medication. With proper instillation counselling and the time gap between medications to reduce this, about 25% of patients waited for 5 min between the two medications. Using an FDCT combination eliminated this issue (17).

The assessment of adherence was conducted using the measurement of bottle weight at the end of the treatment. The mean adherence score of the bottle weight showed a significant difference between the groups. Medication bottle weight as a representative of adherence has been used in other studies, such as in the study of Murdoch et al. in which they used the difference in weight of topical anti-glaucoma given as a representative of the drug used and correlated it with adherence (18). This was in accordance with the study of Hess et al. (19) where adherence to topical medication was measured based on the percentage obtained from the difference in weight of the medication used, similar in our study. The limitation in assessing adherence based on bottle weight is that it can be tampered by the instillation technique, as most patients were of the elderly group, in which manual dexterity control is of concern (20). A more reliable and quantitative method that can be adopted in future studies is the use of electronic devices, such as the Medication Event Monitoring System (21).

Although the results of the RM ANOVA showed a significant reduction in IOP in both the FCDT and NFDT groups, no significant reduction in IOP was found in either group after controlling for the adherence factor. Therefore, adherence is another factor that affects the efficacy of treatment, and it is important to educate patients on compliance and adherence to treatment.

## Conclusion

Both the FCDT and NFDT groups showed a decrease in IOP but more so in FCDT. However, when taking adherence to medication into account, no difference in IOP reduction was found between the groups. Strategies for improving adherence should be combined with treatment methods for better IOP control.

## Figures and Tables

**Table 1:** Adherence score measure by bottle weight of the medication

Adherence score	Percentage of medication used by weight (%)
1	0–20
2	21–40
3	41–60
4	61–80
5	81–100

**Table 2:** Demographic and clinical profile of patients by treatment group (*n* = 55)

Demographic characteristics	FCDT *n* = 29	NFDT *n* = 26	*P*-value
Age (years old)^a^	65.79 (9.5)	69.05 (9.08)	0.146^b^
Ethnicity, *n* (%)			
Malay	24 (82.8)	21 (80.8)	0.100^d^
Chinese	5 (17.2)	5 (19.2)	
Gender, *n* (%)			
Male	21 (72.4)	14 (53.8)	0.153c
Female	8 (27.6)	12 (46.2)	
Systemic illness, *n* (%)			
Hypertension	21 (72.4)	17 (65.4)	0.573c
Diabetes mellitus	10 (36.5)	12 (44.8)	0.378^c^
Ischaemic heart disease	1 (3.4)	4 (15.4)	0.178^d^
Type of glaucoma, *n* (%)			
POAG	25 (86.2)	21 (80.8)	0.721^d^
NTG	4 (13.8)	5 (19.2)	
Laterality of eye, *n* (%)			
Right	20 (69.0)	15 (57.7)	0.386^c^
Left	9 (31.0)	11 (42.3)	
Number of topical pressure lowering drugs, *n* (%)			
Monotherapy	10 (34.5)	5 (19.2)	0.091d
Dual therapy	12 (41.4)	20 (76.9)	
Combination (Xalacom)	7 (24.1)	1 (3.8)	
Type of topical pressure lowering drugs, *n* (%)			
Timolol	12 (41.4)	11 (42.3)	0.944c
Timolol XE	1 (3.4)	6 (23.1)	0.044^d^
Betoxolol	3 (10.3)	0 (0.0)	0.239d
Dorzolamide	6 (20.7)	9 (34.6)	0.239c
Latanaprost	11 (37.9)	16 (61.5)	0.080^c^
Combination (Xalacom)	7 (24.1)	2(7.7)	0.149c

Notes:

a mean (SD);

b Independent *t*-test;

c Pearson’s chi-squared test;

d Fisher’s exact test;

FCDT = fixed-combination dorzolamide/timolol maleate; NFDT = non-fixed combination dorzolamide and timolol XE; NTG = normotensive glaucoma; POAG = primary open-angle glaucoma

**Table 3:** Comparison of IOP based on time (time effect) (*n* = 55)

	FCDT		NFDT	
	
	MD (95% CI)	*P*-value	MD (95% CI)	*P*-value
Baseline-Month 1	4.93 (4.00, 5.86)	< 0.001#	4.92 (4.02, 5.82)	< 0.001#
	4.93 (3.98, 5.88)	< 0.001*	4.92 (4.04, 5.80)	< 0.001*
Baseline-Month 3	5.17 (4.19, 6.15)	< 0.001#	4.85 (3.87, 5.83)	< 0.001#
	5.17 (4.18, 6.16)	< 0.001*	4.85 (3.86, 5.82)	< 0.001*
Month 1-Month 3	0.24 (−0.37, 0.86)	0.978#	−0.08 (−0.73, 0.57)	> 0.950#
	0.24 (−0.37, 0.81)	0.963*	−0.08 (−0.73, 0.57)	> 0.950*

Notes:

# Repeated measures ANOVA analysis was applied followed by pairwise comparison with 95% CI adjustment by Bonferroni correction (*F*(2, 52) = 203.03; *P* < 0.001);

* Repeated measures ANCOVA analysis was applied followed by pairwise comparison with 95% CI adjustment by Bonferroni correction (*F*(2, 51) = 1.01; *P* = 0.341);

Potential covariate (adherence) was controlled by using repeated measures ANCOVA;

MD = mean difference; FCD = Fixed combination dorzolamide/timolol maleate; IOP = intraocular pressure; NFDT = non-fixed combination dorzolamide and timolol XE

**Table 4:** Overall mean difference of IOP among two groups (treatment effect) (*n* = 55)

Comparison	MD (95% CI)	*P*-value
FCDT-NFDT	−1.02 (−2.01, −0.02)	0.046#
	−0.89 (−2.02, 0.25)	0.124*

Notes:

# Repeated measures ANOVA between group was applied (*F* stat (df) = 4.19 (1, 53));

* Repeated measures ANCOVA between group was applied (*F* stat (df) = 2.45 (1, 52));

Potential covariate (adherence) was controlled by using repeated measures ANCOVA;

MD = mean difference; FCDT = fixed combination dorzolamide/timolol maleate; NFDT = non-fixed combination dorzolamide and timolol XE; *P* < 0.05 in considered statistically significant

**Table 5:** Comparison of IOP within among two different treatment groups based on time (time-treatment interaction) (*n* = 55)

	Comparison	MD (95% CI)	*P*-value
Baseline	FCDT-NFDT	−0.90 (−2.12, 0.32)	0.144#
		−0.81 (0.22, −2.26)	0.222*
Month 1	FCDT-NFDT	−0.91 (−1.94, 0.12)	0.081#
		−0.58 (0.32, −1.74)	0.324*
Month 3	FCDT-NFDT	−.22 (−2.38, −0.08)	0.037#
		−1.21 (−0.10, 2.52)	0.070*

Notes:

# Repeated measures ANOVA between group analysis with regard to time was applied (*F*(2, 52) = 0.44; *P* = 0.650);

* Repeated measures ANCOVA between group analysis with regard to time was applied (*F*(2, 51) = 0.90; *P* = 0.412);

Potential covariate (adherence) was controlled by using repeated measures ANCOVA;

MD = mean difference; FCDT = fixed combination dorzolamide/timolol maleate; NFDT = non-fixed combination dorzolamide and timolol XE; *P* < 0.05 in considered statistically significant

**Table 6:** Adherence score (*n* = 55)

	FCDT mean (SD)	NFDT mean (SD)	*P*-value
Bottle weight	78.35 (8.81)	73.29 (9.38)	0.044
Adherence score	4.48 (0.51)	3.92 (0.56)	< 0.001

Notes: Independent *t*-test used; SD = standard deviation; FCDT = fixed combination dorzolamide/timolol maleate; NFDT = non-fixed combination dorzolamide and timolol XE; *P* < 0.05 in considered statistically significant

## References

[1] Ministry of Health (MOH) Malaysia (2017). Clinical practice guidelines: management of glaucoma.

[2] Chew FLM, Salowi MA, Mustari Z, Husni MA, Hussein E, Adnan TH (2018). Estimates of visual impairment and its causes from the national eye survey in Malaysia (NESII). PLoS One.

[3] Tham YC, Li X, Wong TY, Quigley HA, Aung T, Cheng CY (2014). Global prevalence of glaucoma and projections of glaucoma burden through 2040: a systematic review and meta-analysis. Ophthalmology.

[4] Jonas JB, Aung T, Bourne RR, Bron AM, Ritch R, Panda-Jonas S (2017). Glaucoma. Lancet.

[5] Azri NA (2022). Population projection (revised), Malaysia, 2010–2040.

[6] Choudhri S, Wand M, Shields MB (2000). A comparison of dorzolamide-timolol combination versus the concomitant drugs. Am J Ophthalmol.

[7] Kass MA, Heuer DK, Higginbotham EJ, Johnson CA, Keltner JL, Miller JP (2002). The ocular hypertension treatment study: a randomized trial determines that topical ocular hypotensive medication delays or prevents the onset of primary open-angle glaucoma. Arch Ophthalmol.

[8] Hutzelmann J, Owens S, Shedden A, Adamsons I, Vargas E (1998). Comparison of the safety and efficacy of the fixed combination of dorzolamide/timolol and the concomitant administration of dorzolamide and timolol: a clinical equivalence study. International Clinical Equivalence Study Group. Br J Ophthalmol.

[9] Shedden A, Adamsons IA, Getson AJ, Laurence JK, Lines CR, Hewitt DJ (2010). Comparison of the efficacy and tolerability of preservative-free and preservative-containing formulations of the dorzolamide/timolol fixed combination (COSOPT^TM^) in patients with elevated intraocular pressure in a randomized clinical trial. Graefes Arch Clin Exp Ophthalmol.

[10] Diaconita V, Quinn M, Jamal D, Dishan B, Malvankar-Mehta MS, Hutnik C (2018). Washout duration of prostaglandin analogues: a systematic review and meta-analysis. J Ophthalmol.

[11] Francis BA, Du LT, Berke S, Ehrenhaus M, Minckler DS, Cosopt Study Group (2004). Comparing the fixed combination dorzolamide-timolol (Cosopt^®^) to concomitant administration of 2% dorzolamide (Trusopt^®^) and 5% timolol: a randomized controlled trial and a replacement study. J Clin Pharm Ther.

[12] Boyle JE, Ghosh K, Gieser DK, Adamsons IA (1999). A randomized trial comparing the dorzolamide-timolol combination given twice daily to monotherapy with timolol and dorzolamide. Opthalmology.

[13] Strohmaier K, Snyder E, Dubiner H, Adamsons I (1998). The efficacy and safety of the dorzolamide-timolol combination versus the concomitant administration of its components. Dorzolamide-Timolol Study Group. Opthalmology.

[14] Konstas AG, Schmetterer L, Katsanos A, Hutnik CML, Holló G, Quaranta L (2021). Dorzolamide/timolol fixed combination: learning from the past and looking toward the future. Adv Ther.

[15] Konstas AGP, Holló G, Haidich AB, Mikropoulos DG, Giannopoulos T, Voudouragkaki IC (2013). Comparison of 24-hour intraocular pressure reduction obtained with brinzolamide/timolol or brimonidine/timolol fixed-combination adjunctive to travoprost therapy. J Ocul Pharmacol Ther.

[16] Lee NY, Park HYL, Park CK (2016). Effects of a dorzolamide/timolol fixed combination on diurnal intraocular pressure, heart rate, blood pressure, and ocular perfusion pressure in normal-tension glaucoma. Jpn J Ophthalmol.

[17] Holló G, Topouzis F, Fechtner RD (2014). Fixed-combination intraocular pressure-lowering therapy for glaucoma and ocular hypertension: advantages in clinical practice. Expert Opin Pharmacother.

[18] Murdoch I, Nyakundi D, Baker H, Dulku S, Kiage D (2020). Adherence with medical therapy for primary open-angle glaucoma in Kenya—a pilot study. Patients Prefer Adher.

[19] Hess LM, Saboda K, Malone DC, Salasche S, Warneke J, Alberts DS (2005). Adherence assessment using medication weight in a phase IIb clinical trial of difluoromethylornithine for the chemoprevention of skin cancer. Cancer Epidemiol Biomarkers Prev.

[20] Suet Yee KC, Jing Wen L, Chee Tao C, Fun Wee H, Siew Huang L, Chan HK (2018). Adherence and challenges in administering eye medications among glaucoma patients in a Malaysian public tertiary care centre. J Pharm Health Serv Res.

[21] Thompson AC, Woolson S, Olsen MK, Danus S, Bosworth HB, Muir KW (2018). Relationship between electronically measured medication adherence and vision-related quality of life in a cohort of patients with open-angle glaucoma. BMJ Open Ophthalmol.

